# Relationship between MRI First pass Perfusion Parameters and Biventricular Performance in Pulmonary Hypertension

**DOI:** 10.1186/1532-429X-18-S1-P292

**Published:** 2016-01-27

**Authors:** Rebecca E Thornhill, Elena Pena, Lin Yassin Kassab, Carole Dennie, Alexander Dick, Girish Dwivedi, Lisa Mielniczuk

**Affiliations:** 1 grid.412687.e0000000096065108Medical Imaging, The Ottawa Hospital, Ottawa, ON Canada; 2 grid.28046.380000000121822255Radiology, University of Ottawa, Ottawa, ON Canada; 3 grid.34428.39000000041936893XCarleton University, Ottawa, ON Canada; 4 grid.28046.380000000121822255Division of Cardiology, Department of Medicine, University of Ottawa Heart Institute, Ottawa, ON Canada

## Background

The amount of blood flow through the lungs is determined by pulmonary by pressure gradient as well as cardiac function in PH. Dynamic contrast-enhanced MRI (DCE-MRI) enables high temporal resolution imaging to track the first transit of contrast material bolus through the pulmonary circulation. First-pass parameters such as pulmonary transit time (PTT), left ventricular (LV) full width at half maximum (FWHM), and LV time to peak (TTP) have been shown to be impaired in PAH compared to controls. Little has been reported regarding the relationship between first pass perfusion parameters in other groups of PH. Our aim was to explore the relationship between first-pass parameters and RV and LV performance in patients with group I (PAH) and group IV (chronic thromboembolic pulmonary hypertension) PH

## Methods

We prospectively recruited 9 patients with PH (pulmonary arterial hypertension, n=5; chronic thromboembolic pulmonary hypertension, n=4) who underwent CMR at 3T. CMR studies included functional (bSSFP) cine imaging for assessment of ventricular function, as well as phase contrast velocity encoded cine imaging obtained perpendicular to the main pulmonary artery (MPA) for assessment of MPA distensibility (%). To assess PTT, FWHM, and TTP, a 1:10 diluted bolus (0.0025 mmol/kg) of Gadobutrol was injected intravenously at 5 mL/s and followed using a single-shot saturation-recovery gradient-echo sequence in the short axis orientation (at the basal third of both ventricles). First-pass bolus kinetic parameters were determined by sampling the signal intensities in the RV and LV cavities and generating time-intensity curves for each ventricle. PTT was defined as the transit time of blood between the RV and LV, while FWHM and TTP were derived from the shape of the bolus in the LV cavity (Figure). Differences in first-pass parameters between the two groups were assessed using Mann-Whitney U tests and the relationship between each first-pass and functional parameters were investigated using Spearman's rank correlation (rho).

## Results

There were no significant differences in first-pass parameters between groups (P>0.05 for each comparison). Significant negative correlations were found between FWHM and LV ejection fraction (rho=-0.72, p = 0.03) and LV cardiac output (rho=-0.68, p = 0.04) and FWHM was positively correlated with MPA distensibility (rho=0.73, p = 0.02). Trends towards negative correlations between PTT and RV (rho=-0.65, p = 0.06) and LV stroke volume indexed (rho=-0.60, p = 0.09) were also revealed.

## Conclusions

First pass perfusion parameters are similar in Group I and IV patients with PH. An increase in MPA distensibility is associated with an increase in FWHM. This may be attributed to advanced disease when RV cardiac output is decreased resulting in a paradoxical decrease in pulmonary pressures. Increased first-pass bolus dispersion (FWHM) may be a noninvasive marker of impaired pulmonary hemodynamics and biventricular dysfunction in groups I and IV patients with PH.

**Figure 1:**
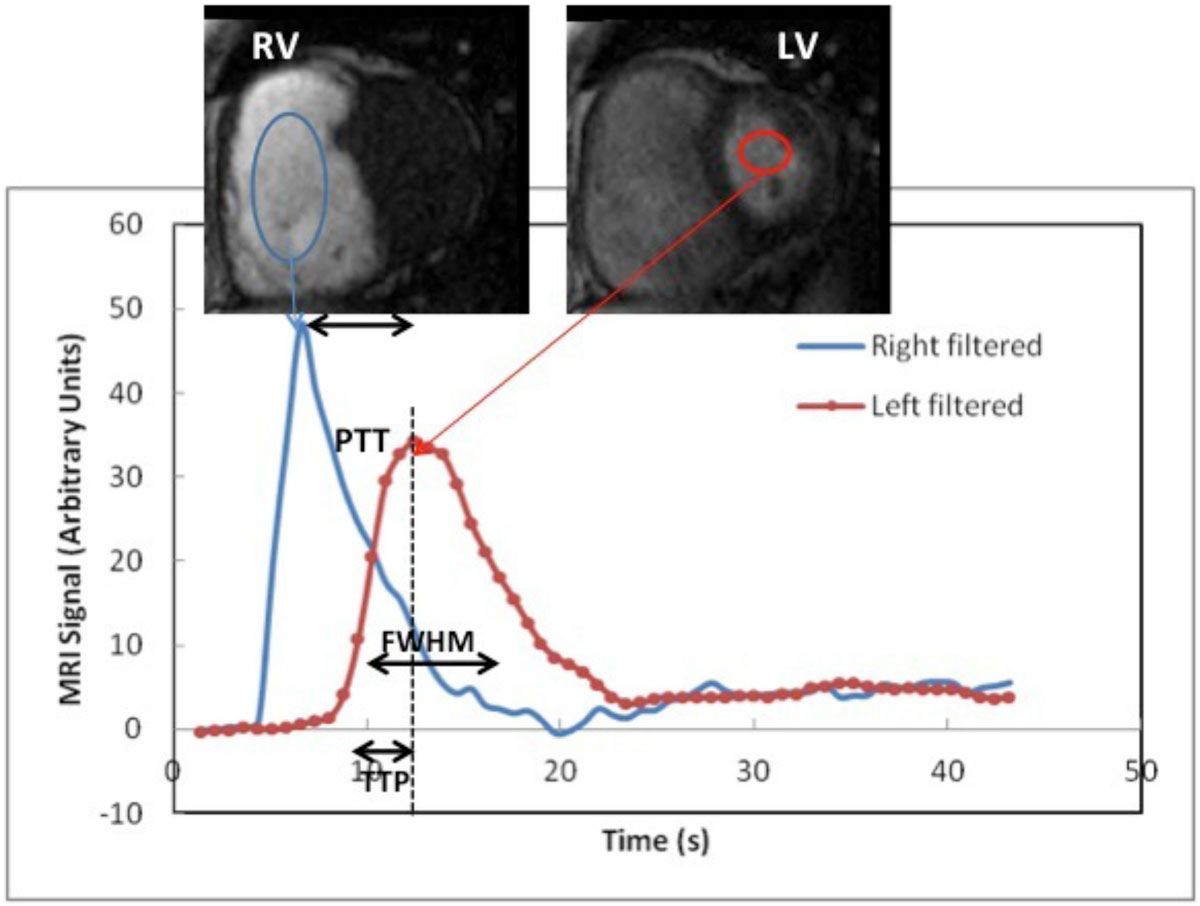
**Time intensity curves show transit of the contrast material bolus through the regions of interest in the right (blue) and left (red) ventricular cavities and indicate the first pass bolus parameters peak to peak PTT, FWHM and TTP**.

